# Triazine and Fused Thiophene-Based Donor-Acceptor Type Semiconducting Conjugated Polymer for Enhanced Visible-Light-Induced H_2_ Production

**DOI:** 10.3390/molecules29122807

**Published:** 2024-06-12

**Authors:** Jian Liu, Shengling Zhang, Xinshu Long, Xiaomin Jin, Yangying Zhu, Shengxia Duan, Jinsheng Zhao

**Affiliations:** 1 College of Agriculture and Bioengineering, Heze University, Heze 274000, China; liujian61@hezeu.edu.cn; 2 Institute of Biotechnology, Chinese Academy of Tropical Agricultural Sciences, Haikou 570100, China; 3 Department of Chemistry and Chemical Engineering, Liaocheng University, Liaocheng 252000, China; zsl20211102@163.com; 4 Department of Chemistry and Engineering, Heze University, Heze 274500, China; longxinshu@sina.com (X.L.); 17661369763@163.com (X.J.); zyy030302@163.com (Y.Z.); 5 CAS Key Laboratory of Photovoltaic and Energy Conservation Materials, Institute of Plasma Physics, Chinese Academy of Sciences, Hefei 230031, China

**Keywords:** conjugated microporous polymer, triazine, fused thiophene, photocatalysis, H_2_ production

## Abstract

Conjugated polymers have attracted significant attention in the field of photocatalysis due to their exceptional properties, including versatile optimization, cost-effectiveness, and structure stability. Herein, two conjugated porous polymers, PhIN-CPP and ThIN-CPP, based on triazines, were meticulously designed and successfully synthesized using benzene and thiophene as building blocks. Based on UV diffuse reflection spectra, the photonic band gaps of PhIN-CPP and ThIN-CPP were calculated as 2.05 eV and 1.79 eV. The PhIN-CPP exhibited a high hydrogen evolution rate (HER) of 5359.92 μmol·g^−1^·h^−1^, which is 10 times higher than that of Thin-CPP (538.49 μmol·g^−1^·h^−1^). The remarkable disparity in the photocatalytic performance can be primarily ascribed to alterations in the band structure of the polymers, which includes its more stable benzene units, fluffier structure, larger specific surface area, most pronounced absorption occurring in the visible region and highly extended conjugation with a high density of electrons. The ΔE_ST_ values for PhIN-CPP and ThIN-CPP were calculated as 0.79 eV and 0.80 eV, respectively, based on DFT and TD-DFT calculations, which revealed that the incorporation of triazine units in the as-prepared CMPs could enhance the charge transfer via S1 ↔ T1 and was beneficial to the photocatalytic decomposition of H_2_O. This study presents a novel concept for developing a hybrid system for preparation of H_2_ by photocatalysis with effectiveness, sustainability, and economy.

## 1. Introduction

Storing abundant solar energy as chemical energy is recognized as a promising pathway to solving the future energy crisis and environmental issues [1]. Hydrogen (H_2_) energy is increasingly being recognized as a viable alternative to traditional fossil fuels, which has the potential to offer practical solutions to current environmental and sustainability challenges [2,3,4,5,6]. Utilizing semiconductors for photocatalytic water splitting to produce H_2_ presents an ideal and potential method for addressing the problem of energy shortage and environmental pollution. Since photocatalysts play the key role in photocatalytic H_2_ analysis, efficient, stable, and economic catalysts have been constantly investigated by a large number of researchers. Inorganic semiconductors have predominantly been utilized as photocatalysts for the production of H_2_ gas, because of their high level of stability and activity [7,8,9,10,11,12]. However, inorganic photocatalysts (such as metal oxides, oxysulfides, sulfides, and nitrides) have certain limitations regarding the low H_2_ evolution efficiency, preparation challenges, limited absorption of visible light, and potentially, the scarcity of their natural resources. Hence, organic semiconductor materials have attracted more and more attentions because of their flexible structural design, diverse synthesis strategies, and adjustable electronic characteristics [13,14,15].

Various organic photocatalysts, such as graphite carbon nitride (g-C_3_N_4_) [16], linear conjugated polymers (CPs) [17], conjugated microporous polymers (CMPs) [18], covalent organic framework (COFs) [19], and covalent triazine framework (CTFs) [20] have been proven to effectively produce H_2_ from H_2_O. Among these, CMPs possess amounts of distinctive features, including excellent light absorptivity, low density, larger surface areas, diversity in synthetic methods, and convenience of modification of photoelectric characteristics, making them attractive as organic photocatalysts. For instance, EL-Mahdy and his coworkers synthesized several kinds of CMPs. TzTz-Py D–A CMP and Cz-TzTz CMP exhibited an impressive HER of 3710 and 15,300 and µmol·g^–1^·h^–1^, respectively [20,21]. Moreover, the Py-ThTh-CMP synthesized by Mohamed and his colleagues exhibited a HER of 1874 µmol·g^–1^·h^−1^ [2].

However, the practical application of CMPs as photocatalysts falls short of requirements due to limitations such as high binding energies of excitons, low carrier mobility migration rate, and limited ranges of light absorption, resulting in its low photocatalytic performance. The development of new photocatalysts with stable structures, visible light responsiveness, and high catalytic efficiency for water splitting has consistently driven the pursuit of increased efficiency in solar-to-hydrogen energy conversion. Recently, there has been a significant focus on donor-acceptor (D-A) type conjugated microporous polymers (CMPs). This is due to the fact that the D-A heterojunction can greatly enhance exciton dissociation and suppress the recombination of photoinduced hole/electron pairs [22,23,24,25]. For example, Wang and his co-workers have prepared pyrene-based polymer (PyP) for photocatalytic H_2_ evolution [26]. Wen and co-workers have developed an efficient photocatalyst for separation of photogenerated charges, based on the Tthiazolo[5,4-d]thiazole (TzTz) system [27]. This catalyst is capable of reducing O_2_ to superoxide radicals and facilitating the subsequent coupling of (arylmethyl)amines, as well as driving H_2_ production using sunlight. Khan et al. described a CMP CNU-TT12.0, which was synthesized via co-polymerization to incorporate ThTh units into a polymeric carbon nitride (PCN) framework. This material exhibited excellent photocatalytic properties for degrading H_2_O, indicating its potential for applications in environmental remediation [28]. However, only a limited number of donor and acceptor units have been utilized in research, such as fluorene, pyrene, phenanthroline, perylene, tetraphenylethene, perylenediimide, aminobenzene, and fused thiophene units as electron donors. Additionally, dibenzothiophene-dioxide, heptazine, 9-fluorenone, benzothiadiazole, diketopyrrolopyrrole, and triazine units have been employed as electron acceptors [29,30,31,32]. Therefore, it remains crucial to continue developing new donor and acceptor units in order to enhance the photocatalytic production of H_2_ from water.

The triazine unit, characterized by its flatness and complete conjugation nature along with strong electronic receptivity, serves as an effective building block for constructing organic photocatalysts to facilitate the photocatalytic analysis of H_2_. Additionally, it can be used as an active site for interface oxidation-reduction reaction due to the hydrophilic nature of nitrogen atoms [33,34,35]. For instance, Han et al. [36] designed and synthesized two covalent trizine frameworks of T3N-CTF and T3H-CTF. The photocatalytic H_2_ production rate of T3N-CTF was 6485.05 μmol·g^−1^·h^−1^, and that of T3H-CTF was 2028.06 μmol·g^−1^·h^−1^. Moreover, Hao et al. [37] successfully synthesized two conjugated microporous polymers with different trizine unit contents by changing the structure of the monomer, and the photocatalytic hydrogen evolution rate (HER) of T-CMP-1 with more trizine units was up to 3214.3 μmol·g^−1^·h^−1^ under visible light irradiation. Additionally, unlike thiophene units linked by the single bonds, fused thiophene is a plane structure with five dimensions and six π electrons, which can provide a certain electron density for the conjugated skeleton, thus promoting the transport of charge carriers [38,39,40]. Ting et al. developed a battery of fused ring-based photocatalysts and showed that increasing the number of fusion rings could efficaciously improve its photo-catalytic properties [38]. The photo-generated electrons and holes in polymers containing both thiophene and triazine units can be effectively separated, thereby greatly accelerating the water-splitting reaction.

Based on the aforementioned discussion, two conjugated porous polymers, PhIN-CPP and ThIN-CPP, containing both trizine units (electron acceptors) and fused thiophene (electron donors) were designed and successfully synthesized for the production of H_2_ through H_2_O degradation. The peripheral unit of the trizine unit in PhIN-CPP is the benzene unit, whereas the peripheral unit of the trizine unit in ThIN-CPP is the thiophene unit. A series of techniques, including ^13^C NMR spectroscopy, scanning electron microscopy (SEM), transmission electron microscopy (TEM), Fourier-transform infrared (FTIR) spectroscopy, Raman spectroscopy, Brunauer–Emmett–Teller (BET) analysis, and photoluminescence (PL) spectroscopy, were employed to characterize structures, surface morphologies, specific surface areas, thermostability, and photoelectric properties. A notable improvement in H_2_ generation activity was observed when the thiophene unit was substituted with a benzene unit. The hydrogen evolution rates (HER) of PhIN-CPP and ThIN-CPP were significantly increased to 5359.92 and 538.49 μmol·g^−1^·h^−1^, respectively, after the addition of a 3wt% Pt cocatalyst. These values are notably higher than the HER observed under catalyst-free conditions. The corresponding results demonstrate that the thiophene unit, acting as bridges in the framework, can function as an electron acceptor and accelerate charge migration in the conjugated polymer, leading to enhanced photocatalytic H_2_ generation activity. Because of the great photocatalytic performance of PhIN-CPP in the reduction of H_2_O to produce H_2_, this study confirms that the selection of a suitable construction monomer remains a promising strategy for preparing high-efficiency photocatalysts.

## 2. Results and Discussion

### 2.1. Characterization of PhIN-CPP and ThIN-CPP

**Figure 1:**
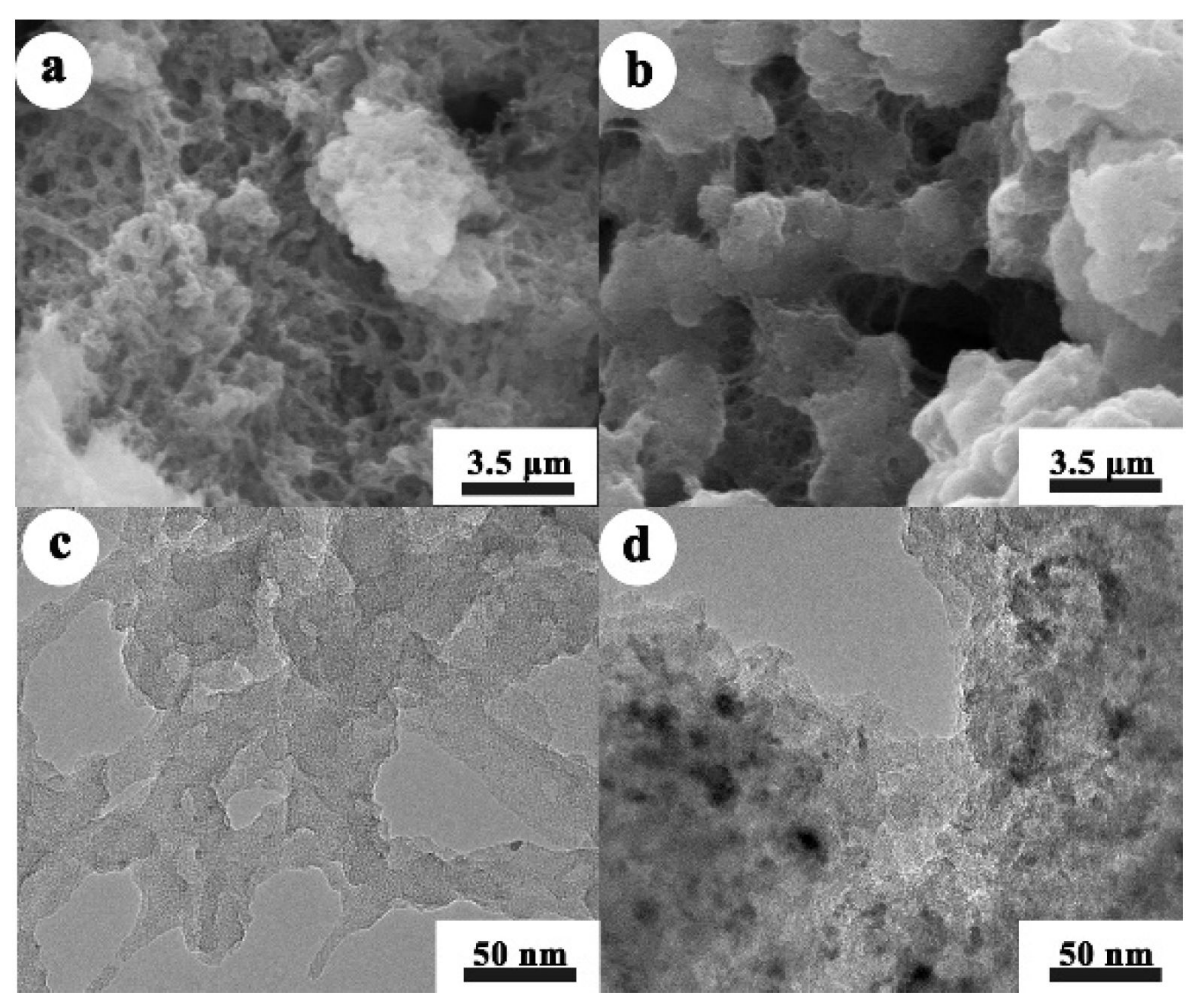
SEM images of PhIN-CPP (**a**) and ThIN-CPP (**b**); TEM images of PhIN-CPP (**c**) and ThIN-CPP (**d**).

**Figure 2:**
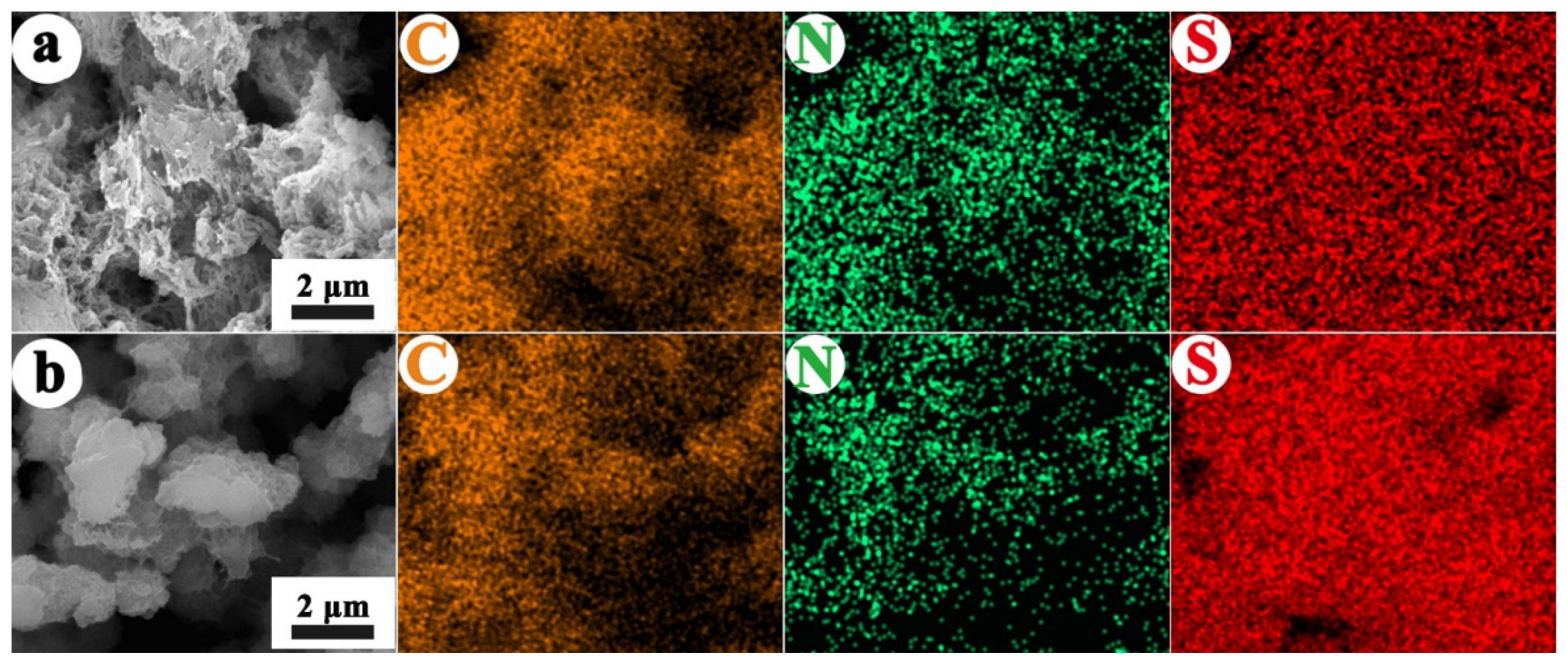
Element mapping images PhIN-CPP (**a**) and ThIN-CPP (**b**).

The morphologies and structures of the PhIN-CPP and ThIN-CPP obtained through the Stille coupling reaction were characterized using SEM and TEM, and illustrated in Figure 1. PhIN-CPP exhibited an alveolate morphology, while ThIN-CPP exhibited a flaky-like morphology. The alveolate morphology of PhIN-CPP was expected to increase the specific surface area and further, offering more active sites for decomposition of water to produce H_2_ gas. The element mapping images of corresponding PhIN-CPP and ThIN-CPP are shown in Figure 2, containing C, N, and S, further confirming the successful synthesis of the two polymers.

**Figure 3:**
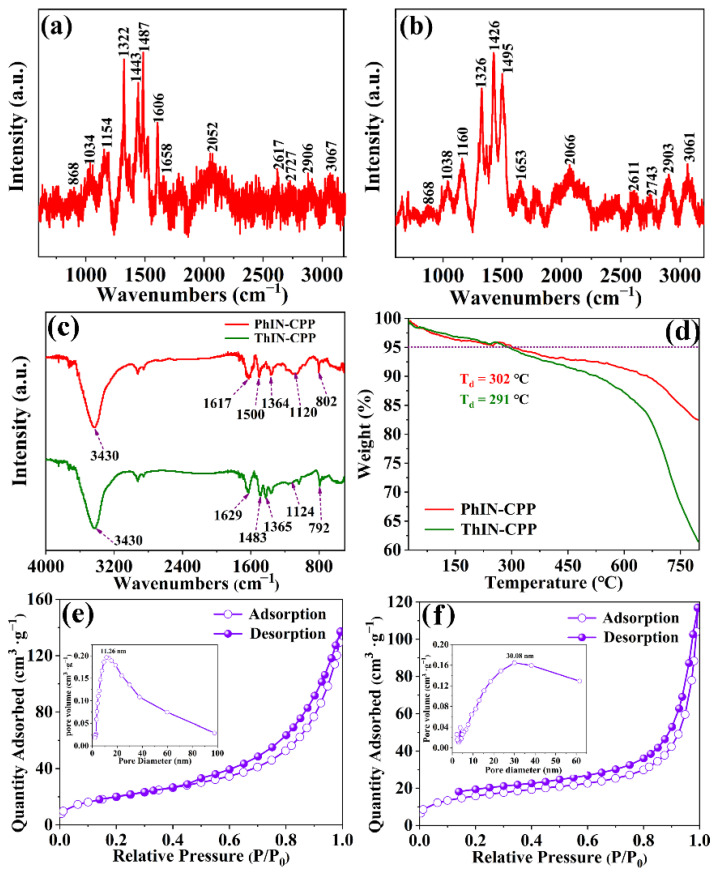
(**a**) Raman spectrum of PhIN-CPP; (**b**) Raman spectrum of ThIN-CPP; (**c**) FT-IR spectra of PhIN-CPP and ThIN-CPP; (**d**) TGA curves of PhIN-CPP and ThIN-CPP; (**e**,**f**) N_2_ adsorption–desorption isotherms of PhIN-CPP and Thin-CPP, respectively. Insert: pore size distribution of the polymers [49]) (a.u. presented as absorbance unit).

As shown in Figure 3a,b, the characteristic bands of PhIN-CPP and ThIN-CPP were present at 1653 and 1658 cm^−1^, respectively, which can be ascribed to the C=N groups in the two polymers. The bands located at 2052 and 2727 cm^−1^ of PhIN-CPP (2066 and 2743 cm^−1^ for ThIN-CPP) can be classified as the stretching vibration of the thiophene ring. The strong bands of PhIN-CPP and ThIN-CPP present at 2617 and 2611 cm^−1^ were related to the S–H stretching vibrations. The bands at 2906 cm^−1^ of PhIN-CPP and at 2903 cm^−1^ of ThIN-CPP were related to the C–C backbone stretching vibration [41]. The bands at 3067 cm^−1^ of PhIN-CPP and 3061 cm^−1^ of ThIN-CPP were related to the O–H backbone stretching vibration [42]. Moreover, other Raman bands located in between the wavenumbers ranging from 1450 cm^−1^–850 cm^−1^ for both PhIN-CPP and ThIN-CPP were assigned to the bending, stretching, and deformation vibrations of C-C bonds and the rocking vibration in the CH_2_ group [43]. Additionally, the bands at 1487 cm^−1^ of PhIN-CPP and 1495 cm^−1^ of ThIN-CPP were related to the C–H in plane vibration.

FT-IR was utilized to analyze the surface organic functional groups of PhIN-CPP and ThIN-CPP. As depicted in Figure 3c, the characteristic high intensity bands at 1617 cm^−1^ and 1629 cm^−1^ of PhIN-CPP and ThIN-CPP were attributed to the vibration of the entire aromatic ring [44,45]. The characteristic high intensity bands of PhIN-CPP at 1500 and 1364 cm^−1^ can be ascribed to the skeleton vibrations of thiophene and triazine rings, respectively [46,47]. The out-of-plane and in-plane stretching vibrations of the C-H bond of PhIN-CPP were observed at 802 cm^−1^ and 1120 cm^−1^, respectively. [48]. The skeletal vibrations of the thiophene and triazine rings in ThIN-CPP were observed at 1483 cm^−1^ and 1365 cm^−1^, respectively. Additionally, the out-of-plane and in-plane stretching vibrations of the C-H bond were detected at 792 cm^−1^ and 1124 cm^−1^, respectively. These findings are consistent with the characteristic vibrational modes of the compound [48]. Furthermore, the broad absorption peak at 3430 cm^−1^ in both samples was attributed to the stretching vibrations of –OH, which resulted from the adsorption of H_2_O molecules during the testing procedure.

In addition, the thermal decomposition processes of PhIN-CPP and ThIN-CPP were investigated at a heating rate of 5 °C·min^−1^ from 20 to 800 °C under a nitrogen atmosphere, as shown in Figure 3d. Both complexes, PhIN-CPP and ThIN-CPP, exhibited similar thermal behavior and underwent two stages of weight loss. The first stage of weight loss for PhIN-CPP (observed: 21.90%) occurred from 20 to 302 °C, while for ThIN-CPP (observed: 12.80%), it occurred from 20 to 291 °C, corresponding to the loss of water molecules. Subsequently, the second continuous weight loss occurred above 302 °C for PhIN-CPP and at 291 °C for ThIN-CPP due to the decomposition of organic ligands and backbone collapse of the coordination polymers. It was observed that PhIN-CPP had less weight loss than ThIN-CPP under the same heat treatment process, indicating better thermal stability for PhIN- CPP compared to ThIN-CPP. Furthermore, the XRD patterns of these two polymers did not show any obvious diffraction peaks, indicating that they have an amorphous structure, as shown in Figure S2.

Generally, the specific surface area of photo-catalysts has a significant impact on their photocatalytic activity. Therefore, N_2_ adsorption-desorption experiments were conducted to determine the specific surface area and pore size distribution of polymers. The BET surface area of PhIN-CPP and ThIN-CPP were found to be 73.84 cm^2^ g^−1^ and 55.48 cm^2^ g^−1^, respectively, exhibiting type IV isotherm with small hysteresis, as shown in Figure 3e,f. Furthermore, the inserted figures illustrate the pore size distribution of the polymer, showing relatively wide pore distributions. The pore size of PhIN-CPP was mainly concentrated at 11.26 nm, while that of ThIN-CPP was mainly concentrated at 30.08 nm. Additionally, PhIN-CPP has a larger specific surface area and rich mesopores compared to Thin-CPP, providing more active sites for the reduction reaction on the surface of the photocatalyst and further improving its photocatalytic efficiency.

**Figure 4:**
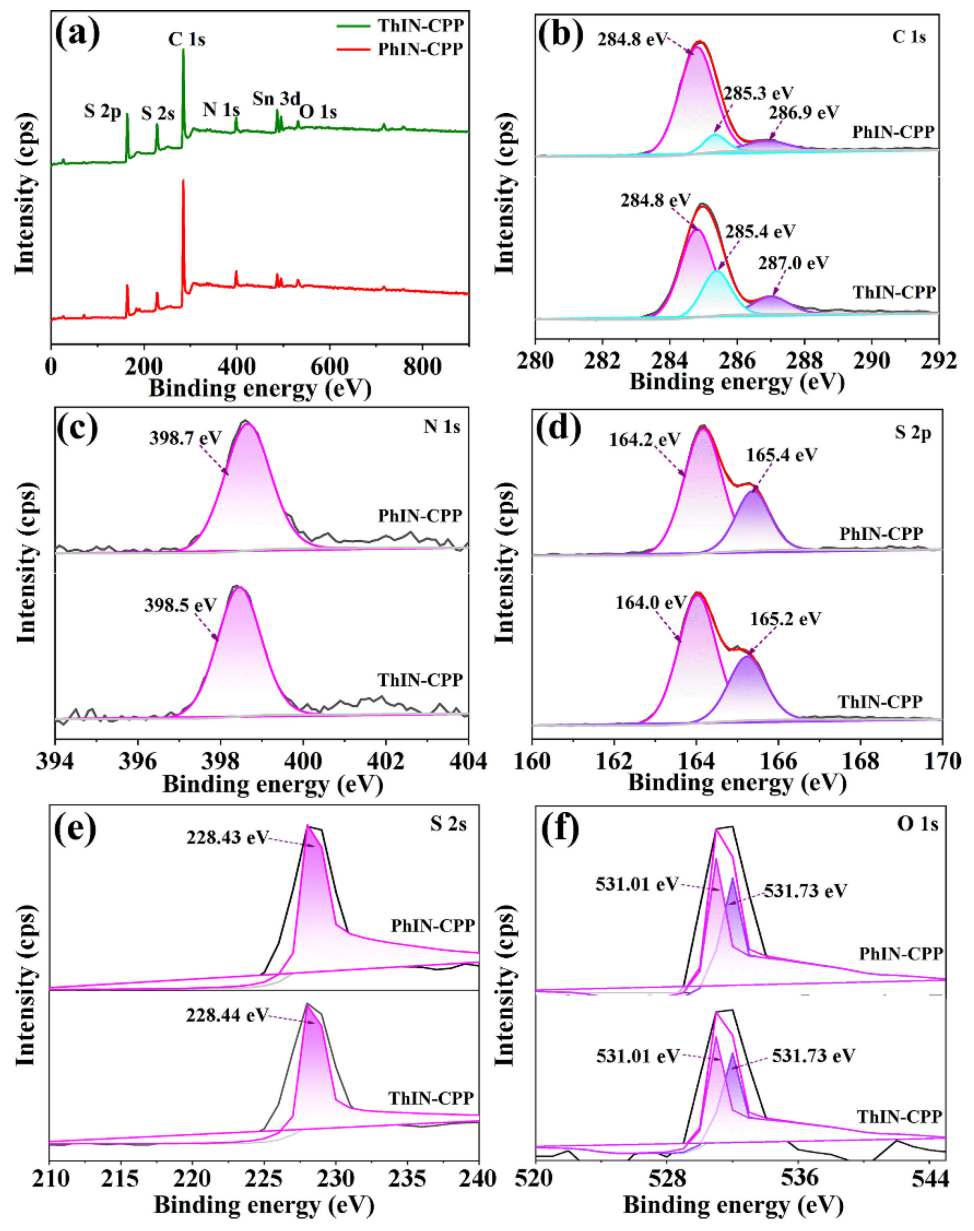
(**a**) XPS survey spectra of PhIN-CPP and ThIN-CPP; (**b**) high resolution of C 1s spectra; (**c**) high resolution of N 1s spectra; (**d**) high resolution of S 2p spectra; (**e**) high resolution of S 2s spectra; and (**f**) high resolution of O 1s spectra.

XPS studies were conducted to confirm the compositions of the two polymers. The total scans of XPS spectra clearly indicated the coexistence of C, N, and S elements in both PhIN-CPP and ThIN-CPP, as illustrated in Figure 4. The C1s spectrum of PhIN-CPP (Figure 4b) was analyzed, and peak positions were observed at 284.8 and 286.9 eV, which corresponded to C-C and C-N, respectively. Similarly, the C1s spectrum of ThIN-CPP (Figure 4b) was fitted with the peaks at 284.8 and 287.0 eV, corresponding to C-C and C-N, respectively. The N1s spectra of PhIN-CPP and ThIN-CPP (Figure 4c) were fitted with the peaks at 399.8 eV and 398.5 eV, respectively, which can be ascribed to nitrogen atoms of triazine rings. The S 2p spectrum of PhIN-CPP (Figure 4d) was fitted with the peaks at 164.2 and 165.4 eV, which can be described by the characteristic doublets of S 2p_3/2_ and S 2p_1/2_, respectively. Similarly, the S 2p spectrum of ThIN-CPP was fitted with the peaks at 164.0 and 165.2 eV, corresponding to S 2p_3/2_ and S 2p_1/2_, respectively. The S 2s spectra of PhIN-CPP and ThIN-CPP (shown in Figure 4e) were fitted with the peaks at 228.43 eV and 228.44 eV, respectively. Moreover, The O 1s spectra of PhIN-CPP and ThIN-CPP (Figure 4f) were fitted with the peaks at 531.01 and 531.73 eV, ascribed to O^2−^ and –OH groups, respectively. Furthermore, the distinctive peaks of Sn 3d can also be observed in the XPS spectra, suggesting the presence of residual Sn compounds in the sample. All these results clearly indicated the successful synthesis of the two polymers.

### 2.2. Visible Light Response and Energy Band Analysis of Polymers

**Figure 5:**
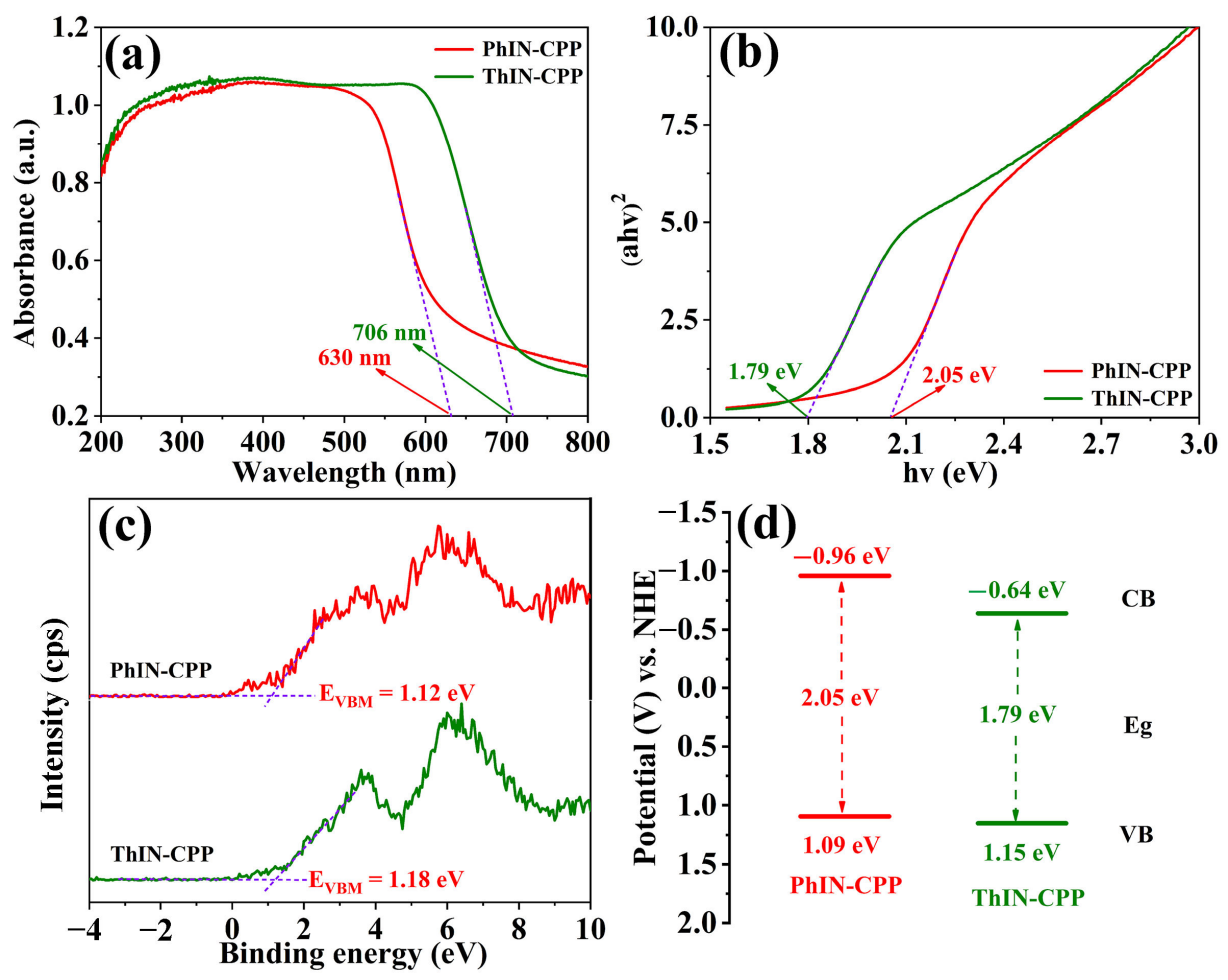
(**a**) UV diffuse reflection spectrum of PhIN-CPP and ThIN-CPP; (**b**) band gap diagram of PhIN-CPP and ThIN-CPP; (**c**) VB-XPS spectrum of PhIN-CPP and ThIN-CPP; (**d**) energy band structure diagrams of PhIN-CPP and ThIN-CPP (a.u. presented as absorbance unit and the purple dashed lines correspond to the tangent of the curve).

The light absorption properties of two polymers were studied by ultraviolet visible diffuse reflection spectroscopy. As shown in Figure 5, both polymers showed strong light absorption capacity across the entire visible light range. In comparison to PhIN-CPP, a more pronounced red-shift trend of Thin-CPP can be observed in the light absorption range. This can be attributed to the enhanced electronic properties of thiophene cells [46,50]. Furthermore, the photonic band gaps of PhIN-CPP and Thin-CPP were calculated as 2.05 eV and 1.79 eV [51], respectively (Figure 5b). This calculation was based on the absorption edge of the ultraviolet visible diffuse reflection spectrum, indicating wide light absorption and a narrow band gap. These characteristics are further beneficial for driving photocatalytic hydrogen production. Moreover, the energy bandgap (1.79 eV) of ThIN-CPP was smaller than that of PhIN-CPP (2.05 eV), which was mainly ascribed to the differences in their structures. The Br and S in 2,4,6-tribromo-3-(5-bromothiophen-2-yl)pyridine are located in the adjacent position. When they undergo electrophilic substitution with Sn compounds, the resulting ThIN-CPP steric hindrance was increased, leading to higher energy levels. This further resulted in a smaller energy difference between the valence band (VB) and conduction band (CB). Moreover, compared to ThIN-CPP, the PhIN-CPP exhibited a smaller spatial steric effect, leading to an increase in band gap of 2.05 eV. Furthermore, the calculation of absorption coefficient (A) was described as follows. The calculation of absorption coefficient was based on Tauc’s equation [52]:

(1) $$\alpha h\upsilon =A\;{\left(h\upsilon -E_{g}\right)}^{n/2}$$

where *α* is the absorbance, *h* is the Planck constant (4.136 × 10^−15^ eV·s), *A* is the absorption coefficient. The value of n depends on the inherent properties of the material itself and whether the transition semiconductor is indirect (*n* = 4) and direct (*n* = 1). Herein, the value of *n* is 1. Moreover, the obtained values of absorbance for PhIN-CPP and ThIN-CPP correspond to the absorbance at 630 nm and 706 nm, respectively. Thus, the *A* of the samples could be evaluated through the slope of the tangent in the plots of (*αhv*)^2^ vs. photon energy (*hv*) owing to PhIN-CPP and ThIN-CPP being a direct bandgap semiconductor. Therefore, the A was calculated to be 4.44 and 4.17 for PhIN-CPP and ThIN-CPP, respectively, indicating that the resulting polymers were donated as excellent light-absorbing materials.

In addition, the maximum valence bands (*E_VBM_*) of PhIN-CPP and ThIN-CPP were calculated as 1.12 eV and 1.18 eV, respectively, as can be seen from Figure 5c. The valence band value (VB) relative to the standard hydrogen electrode can be calculated using the following equation:

(2) $$E_{VB}vs.\;NHE=\varphi +E_{VBM}-4.5$$

in which *φ* represents the power function of the testing instrument, (*φ* = 4.47 eV vs. vacuum). Therefore, the VB values of Phin-CPP and Thin-CPP were calculated as 1.09 eV and 1.15 eV vs. *NHE*, respectively [53,54,55]. Moreover, the guide band values (CB) of PhIN-CPP and ThiN-CPP were calculated by the equation:

(3) $$E_{CB}=E_{VB}-E_{g}$$

The *CB* values of PhIN-CPP and ThIN-CPP were determined to be −0.96 eV and −0.64 eV vs. NHE, as shown in Table 1, according to the aforementioned calculation. The band diagrams of the two polymers are illustrated in Figure 5d. It was evident that the CB energy levels of both PhIN-CPP and ThIN-CPP were significantly higher than the reduction potential of H^+^/H_2_ (0.00 eV vs. NHE), providing sufficient impetus for H_2_ evolution.

### 2.3. Photocatalytic H_2_ Production Properties of PhIN-CPP and ThIN-CPP

**Figure 6:**
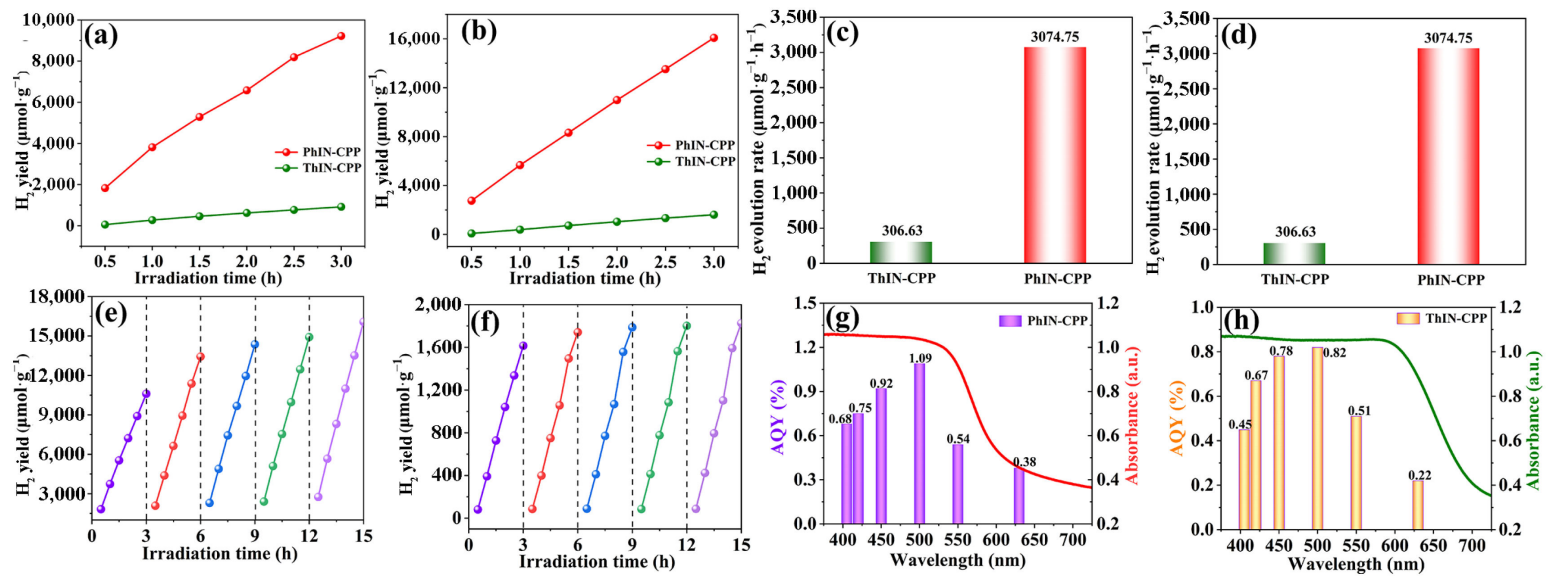
(**a**) Photocatalytic H_2_ yield (μmol·g^−1^) of PhIN-CPP and ThIN-CPP without any catalyst promoter under visible light. (**b**) Photocatalytic H_2_ yield (μmol·g^−1^) of PhIN-CPP and ThIN-CPP under visible light with 3wt% Pt catalyst promoter. (**c**) HER of PhIN-CPP and ThIN-CPP without any catalyst promoter under visible light. (**d**) HER of PhIN-CPP and ThIN-CPP with 3wt% Pt catalyst promoter under visible light. (**e**) PhIN-CPP and (**f**) ThIN-CPP photocatalytic H_2_ precipitation stability test within 15 h of five cycle experiments (the deep purple line, red line, blue line, green line, and light purple line respectively represented the stability test within 15 h of five cycle experiments (1–5)). (**g**) AQY of PhIN-CPP and (**h**) AQY of ThIN-CPP.

To investigate the photocatalytic H_2_ production performance of the prepared materials, a photocatalytic H_2_ production experiment was conducted under visible light (λ > 420 nm) using triethanolamine (TEOA) as the sacrificial agent and 1-methyl-2-pyrrolidone (NMP) as the water-soluble solvent. Figure 6a,b illustrate the photocatalytic H_2_ production process of PhIN-CPP and ThIN-CPP for 3 h without Pt promoter, respectively. As the illumination time was extended, the H_2_ output showed an almost linear increase, indicating that both polymers exhibited good stability in photocatalytic H_2_ production. Without any catalyst promoter, the photocatalytic H_2_ rates (HER) of PhIN-CPP and THIN-CPP polymers were 3074.75 and 306.63 μmol·g^−1^·h^−1^, respectively (Figure 6c). However, the HER rates of PhIN-CPP and ThIN-CPP were up to 5359.92 and 538.49 μmol·g^−1^·h^−1^, respectively, with the addition of 3wt% Pt promoter (Figure 6d). The disparity in the photocatalytic H_2_ production activity of the two polymers can be attributed to their distinct structures. The substitution of the thiophene unit with the phenyl unit has been found to effectively prolong the recombination of photogenerated carriers and promote effective separation and transfer of such carriers, ultimately resulting in a higher H_2_ production rate.

The HER rates of other materials are listed in Table 2 in order to better elucidate the photocatalytic performance of polymers in comparison with other materials in the aspect of HER. It can be observed that the HER of Py-TP-BTDO was up to 115,030 μmol·g^−1^·h^−1^, surpassing the performance of the polymers obtained in this study, which can be ascribed to the efficient separation of light-generated electrons/holes due to the definite D-*π*-A structure and the broad light absorption range [56]. Moreover, the DBC-BTDO-2 synthesized by Zhang et al. showed a high HER of 301,920 μmol·g^−1^·h^−1^, much higher than that of PhIN-CPP (5359.92 μmol·g^−1^·h^−1^) and ThIN-CPP (538.49 μmol·g^−1^·h^−1^) in this study. The high HER of DBC-BTDO-2 can be ascribed to the abundant sulfone groups in BTDO, serving as the electron-output center to reduce protons to hydrogen gas [57]. Furthermore, in comparison with other polymer materials, the polymers obtained in this study exhibited moderate hydrogen production performance. However, compared to other materials, such as g-C_3_N_4_/WS_2_ (101 μmol·g^−1^·h^−1^) [58], PBN (223.5 μmol·g^−1^·h^−1^) [59], NDI-BTzF-PS-PEG-COOH (634.6 μmol·g^−1^·h^−1^) [60], and WS_2_-WO_3_·H_2_O/g-C_3_N_4_ (1276.9 μmol·g^−1^·h^−1^) [61], polymer materials showed a significant advantage in H_2_ production. The remarkable disparity in the photocatalytic performance can be primarily ascribed to alterations in the band structure of the polymers, which include its more stable benzene units, fluffier structure, larger specific surface area, most pronounced absorption occurring in the visible region, and highly extended conjugation with a high density of electrons. The results clearly indicated that polymer materials can be used for H_2_ production with high effectiveness, sustainability, and economy.

Additionally, investigations were conducted to assess the stabilities of PhIN-CPP and ThIN-CPP in photocatalytic H_2_ production. The corresponding results are presented in Figure 6e,f, respectively. The H_2_ production capacity of five cycles within 15 h still exhibited an increasing trend, suggesting that the prepared PhIN-CPP and ThIN-CPP demonstrate good stability in H_2_ production. Additionally, the apparent quantum yield (AQY) magnitude is a crucial parameter for characterizing the photocatalytic performance of reactive polymers. In this study, the AQY of PhIN-CPP and Thin-CPP was investigated under monochromatic light irradiation using various bandpass filters with different wavelengths. The results are presented in Figure 6g,h. The AQY values of 0.68%, 0.75%, 0.92%, 1.09%, 0.54%, and 0.38% for PhIN-CPP corresponded to 405, 420, 450, 500, 550, and 630 nm, respectively, while the AQY values of 0.45%, 0.67%, 0.78%, 0.82%, 0.51%, and 0.22% for Thin-CPP corresponded to 405, 420, 450, 500, 550, and 630 nm were, respectively. The highest AQY of PhIN-CPP and Thin-CPP at 500 nm was up to 1.09% and 0.82%, respectively. However, the AQY at 630 nm was only 0.38% and 0.22%, respectively, which can be ascribed to the weak light absorption capacity at 630 nm. Furthermore, the AQY values of PhIN-CPP and ThIN-CPP were highly consistent with the changes in light absorption intensity observed in the UV diffuse reflection spectrum. There were no variations observed among the UV diffuse reflection spectra, FT-IR, XRD, and SEM images before and after the PhIN-CPP photocatalytic H_2_ production cycle experiment. This confirms the high structural stability of the polymer (Figures S3 and S4).

### 2.4. Investigations on the Generation and Separation of Photovoltaic Carriers of Polymers

**Figure 7:**
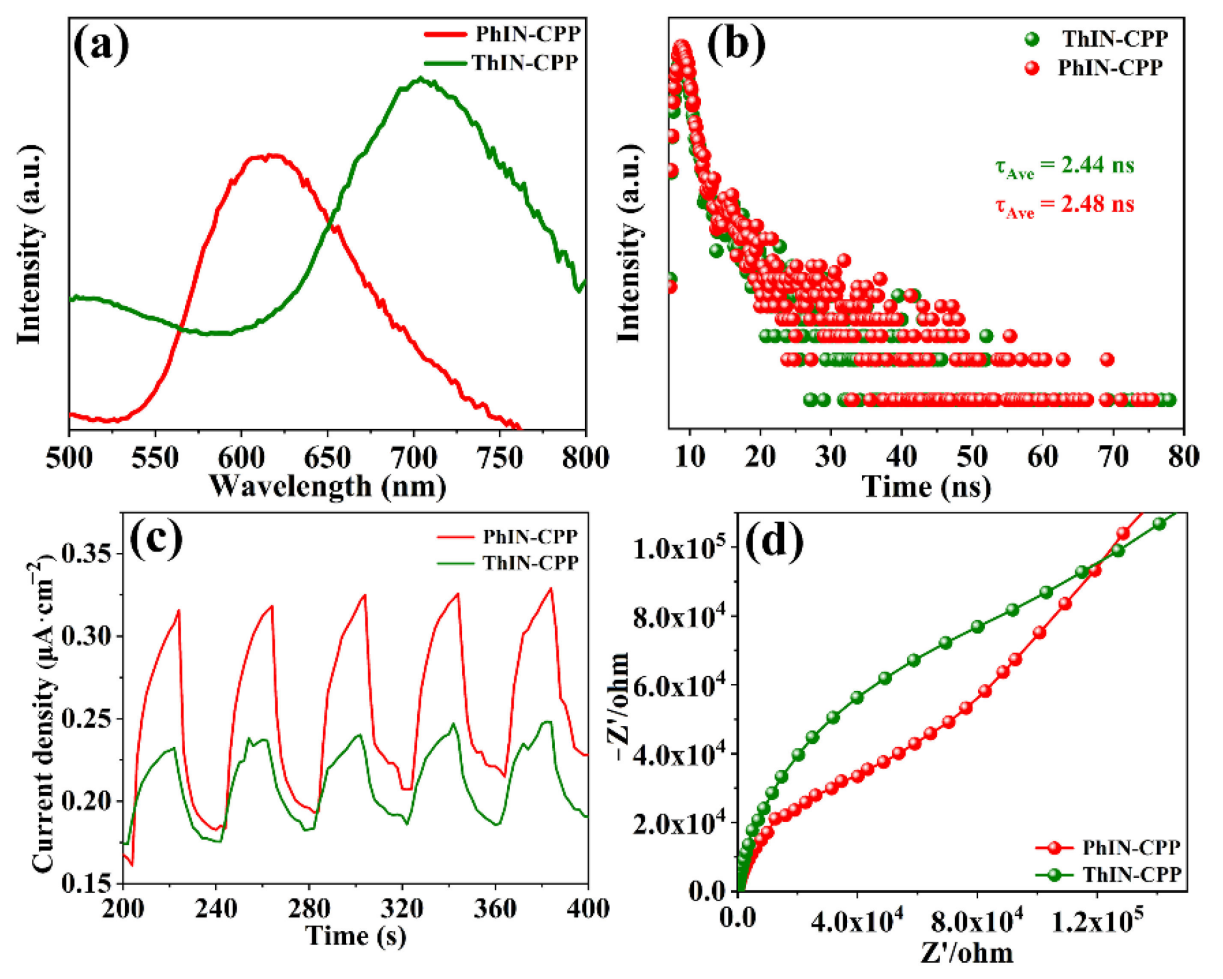
(**a**) The photoluminescence (PL) spectrum of the polymer; (**b**) the time-resolved PL spectrum of the polymer; (**c**) the transient photocurrent response diagram of the polymer; (**d**) the electrochemical impedance diagram of the polymer (a.u. presented as arbitrary unit).

Generally, photoluminescence (PL) spectroscopy is commonly employed to assess the separation efficiency of photogenerated charges in photocatalysts. The corresponding PL spectra of PhIN-CPP and ThIN-CPP are presented in Figure 7a. The photoluminescence (PL) intensity of PhIN-CPP was significantly reduced compared to that of ThIN-CPP. This indicated that the substitution of thiophene units with benzene units in the connection unit of triazine can effectively inhibit the recombination of photogenerated charges in polymers, thereby improving the photocatalytic performance. The time-resolved PL spectra of the two polymers are shown in Figure 7b. The average lifespan of ThIN-CPP’s PL was 2.44 ns, while the average lifespan of PhIN-CPP’s PL was slightly longer at 2.48 ns. The extension of carrier lifetime was advantageous for the capture of reactants and the delay of charge recombination [36]. Transient photocurrent response and electrochemical impedance spectrum (EIS) are also effective methods for investigating the charge separation efficiency of photocatalysts. As shown in Figure 7c, a negligible amount of photocurrent was observed in the dark, while two polymers prepared in this study exhibited a quick photocurrent response. Furthermore, both polymers demonstrated a relatively stable photocurrent signal when exposed to light. The photocurrent intensity of PhIN-CPP was observed to be higher than that of ThIN-CPP, indicating that PhIN-CPP has the capability to effectively utilize more visible light in order to generate and transfer a greater number of photo-generated carriers (Figure 7c). Electrochemical impedance spectroscopy (EIS) results, as shown in Figure 7d, could demonstrate the charge transfer rate in the absence of light. Notably, PhIN-CPP exhibited a smaller arc radius and lower resistance in charge transfer compared to ThIN-CPP, suggesting that the charge transfer efficiency of PhIN-CPP was higher than that of ThIN-CPP after replacing the thiophene unit with the benzene unit. This finding is consistent with the results of photocatalytic H_2_ production [67].

### 2.5. Electron Paramagnetic Resonance (EPR) Analysis

**Figure 8:**
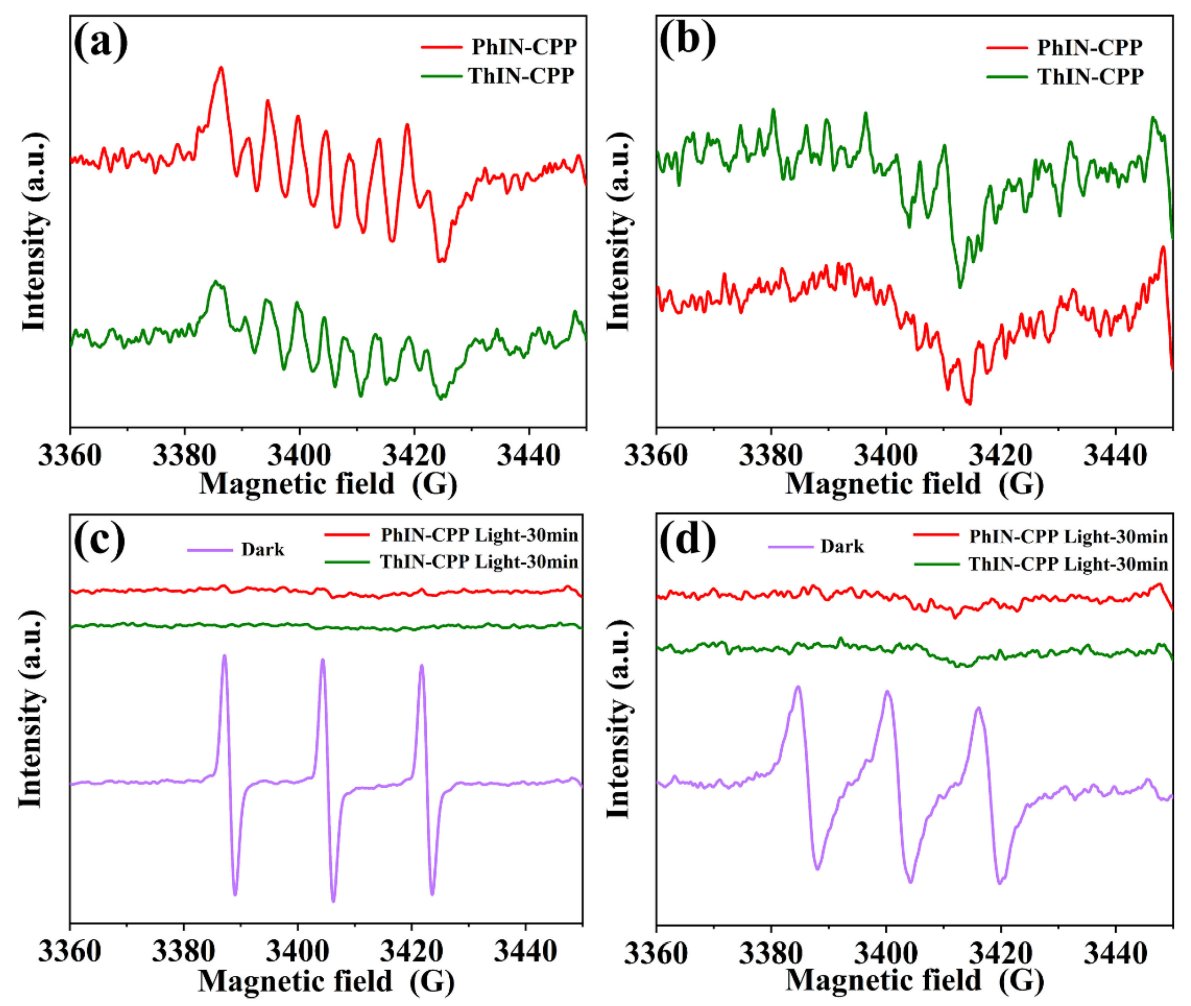
(**a**) DMPO-·O_2_^−^ signal of PhIN-CPP and ThIN-CPP in methanol solution; (**b**) DMPO^−^·OH signal of PhIN-CPP and ThIN-CPP in H_2_O solution; (**c**) EPR diagram of PhIN-CPP and Thin-CPP detecting photoelectrons in in H_2_O solution; (**d**) EPR diagram of PhIN-CPP and ThIN-CPP detecting photogenerated hole in acetonitrile solution (a.u. presented as arbitrary unit).

To gain a deeper understanding of the redox ability of photocatalysts, electron paramagnetic resonance (EPR) analysis was employed to investigate the types of free radicals produced on the surface of polymers. Herein, DMPO was applied as a free radical capture agent to capture ·O_2_^−^ and ·OH generated on the surface of photocatalysts in aqueous solution. As shown in Figure 5d, the conduction band values of PhIN-CPP (−0.96 eV vs. NHE) and Thin-CPP (−0.64 eV vs. of NHE) were much smaller than those of O_2_/·O_2_^−^ (−0.33 eV NHE), suggesting that the electrons in the polymer conduction can reduce the dissolved O_2_ to·O_2_^−^ (O_2_+ e^−^ →·O_2_^−^) [68]. All polymers displayed an obvious DMPO-·O_2_^−^ signal (Figure 8a), and the peak intensity of DMPO-·O_2_^−^ signal in PhIN-CPP was found to be higher in comparison to that in ThIN-CPP, suggesting that PhIN-CPP can produce more ·O_2_^−^ and further has higher reduction activity. Additionally, the valence band values of PhIN-CPP (1.09 eV vs. NHE) and ThIN-CPP (1.15 eV vs. NHE) were significantly lower than the oxidation potential of OH^−^/·OH (1.99 eV vs. NHE), indicating that the holes generated in the polymer valence band were unable to oxidize OH^−^ to ·OH. As a result, no DMPO-·OH signals were detected in either of the two polymers (Figure 8b) [69].

Additionally, the EPR experiment was further applied to directly detect phototonus electrons and holes in the photocatalytic environment. 2,2,6,6-Tetramethylpyridine oxide (TEMPO) is a typical spin-labeled molecule, which can be used to capture photoelectrons (e^−^) and holes (h^+^) produced on the surface of PhIN-CPP and Thin-CPP. As shown in Figure 8c,d, the characteristic peak of TEMPO can be observed in both water solution acetonitrile solution in the absence of light. After 30 min of irradiation, the intensity of the characteristic peak of TEMPO decreased significantly, which could be ascribed to the conversion of TEMPO to TEMPOH and TEMPO^+^ in water solution and acetonitrile solution, respectively [70]. The above results strongly prove that PhIN-CPP and ThIN-CPP can produce electrons (e^−^) and holes (h^+^) under light irradiation.

### 2.6. DFT and TD-DFT Calculations for PhIN-CPP and ThIN-CPP

**Figure 9:**
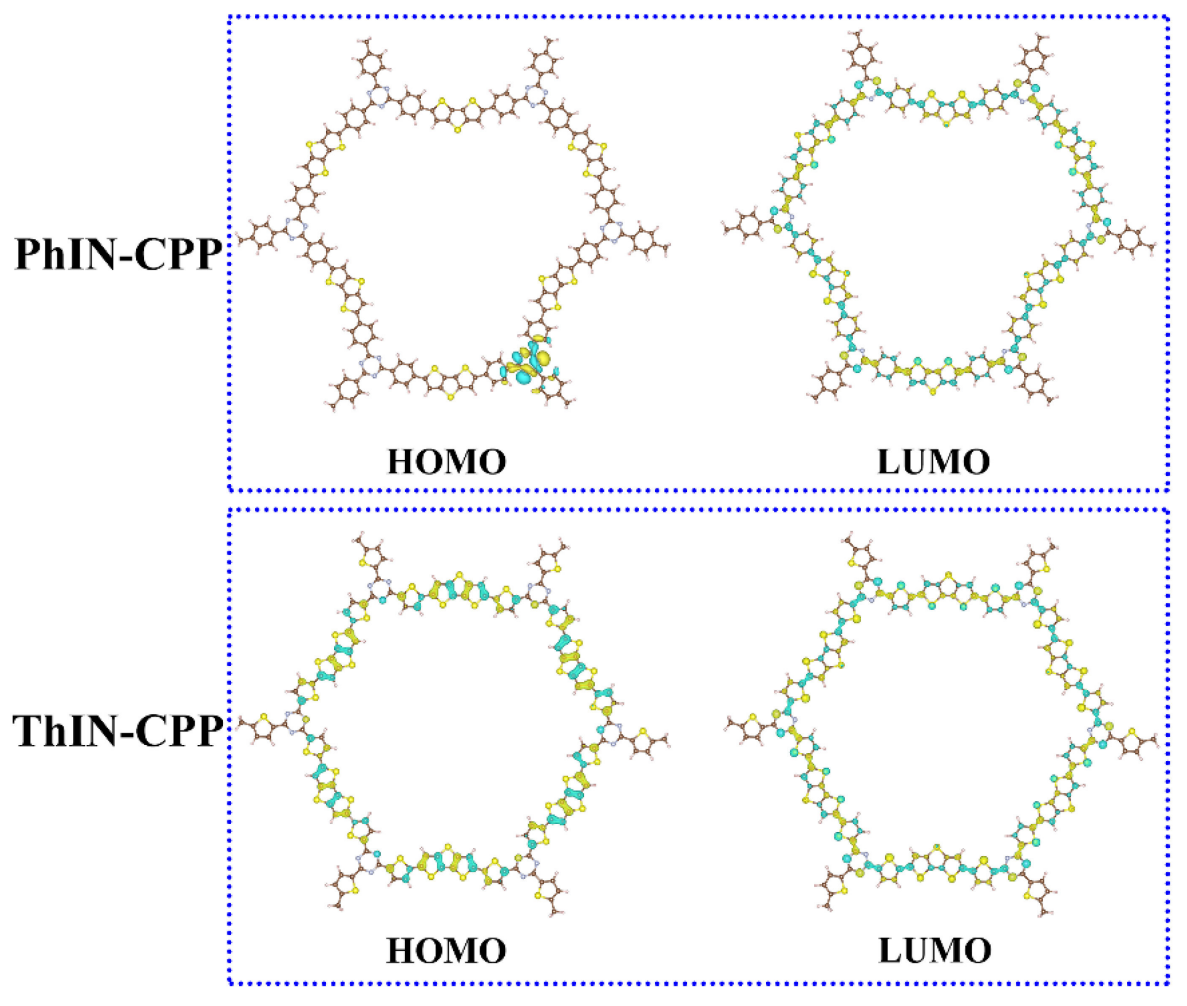
The HOMO and LUMO plots for two ring structures, where the yellow and cyan colors are positive and negative orbital phases whose isovalue is 0.02.

To explain the crucial role of absorbed light in enhancing the efficiency of H_2_ production in prepared compounds at their excited states, the electronic structures for studied molecules were investigated by density functional theory (DFT). The ring structures were optimized by extended tight binding (xTB) program by GFN1 method [71], and the fragments of ring structures were optimized by Gaussian 16 C.02 [72] at B3LYP [73,74] functional with Grimme’s DFT-D3(BJ) [75] empirical dispersion correction and def2-SVP [76,77] level of theory. For S1 and T1, excited states for studied molecules were calculated at time-dependent DFT (TD-DFT) with the TD-B3LYP-D3(BJ)/def2-TZVP level of theory [78,79]. Moreover, the HOMO (highest occupied molecular orbital), LUMO (lowest unoccupied molecular orbital), electron and hole were calculated by Multiwfn 3.8 (dev) [80] and plotted by VESTA 3.5.5 [81]. As shown in the natural transition orbitals (NTOs) analysis (shown in Figure 9), PhIN-CPP demonstrated a delocalized S1 state, while ThIN-CPP exhibited a localized π-π* transition on thiophene fragments, which can also explain the better HER photocatalytic activity of PhIN-CPP in comparison to that of ThIN-CPP.

**Figure 10:**
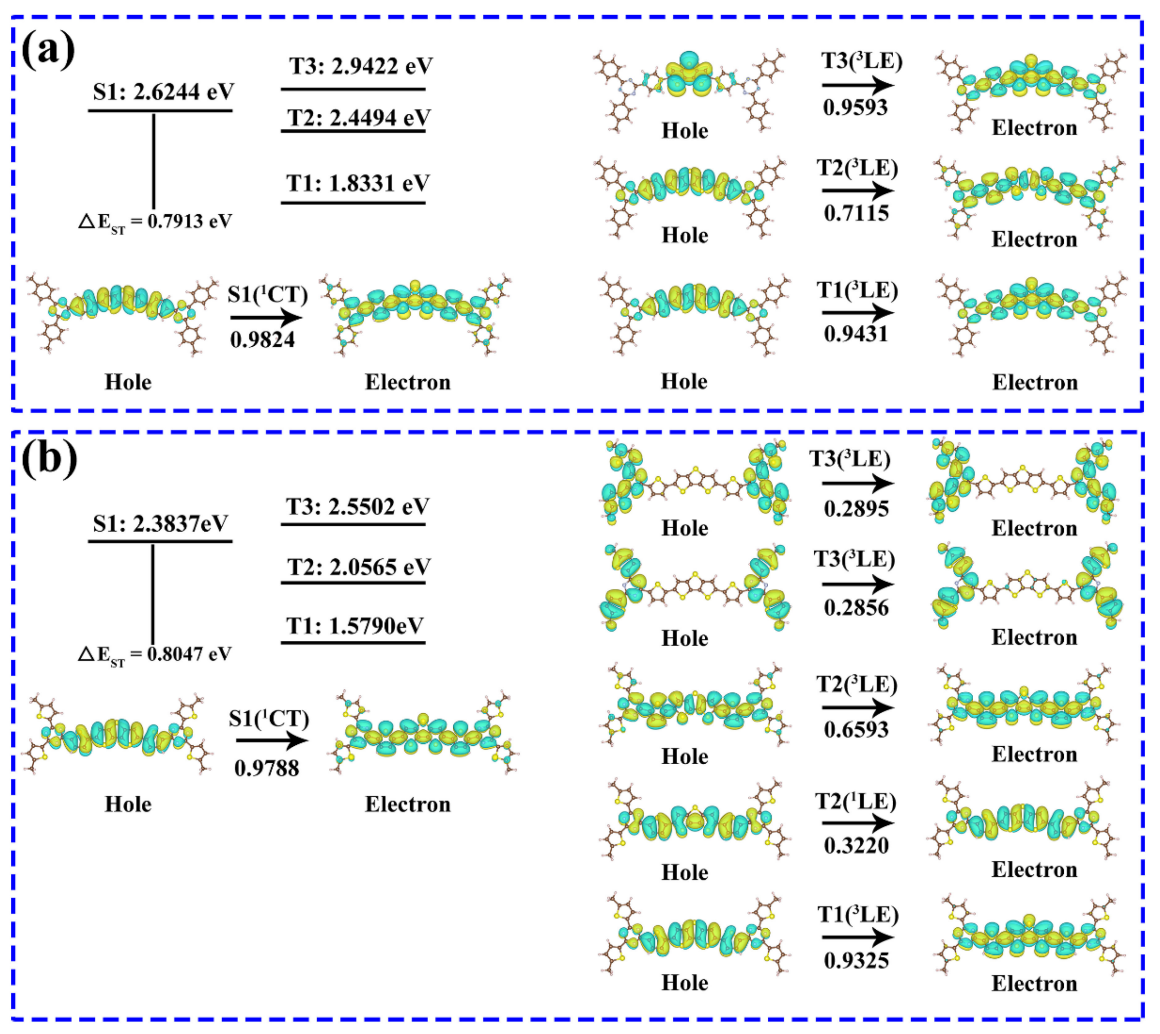
Natural transition orbital characteristics of S1 and Tn (n = 1–3) excited states of (**a**) extracted structure of PhIN-CPP and (**b**) extracted structure of ThIN-CPP, whose isovalue is 0.01.

It is known that the small energy gap (ΔE_ST_) between the singlet S1 and triplet T1 levels of nitrogen-containing organic materials can enhance intermolecular charge transfer transitions and spin-orbit coupling effects [82,83]. Therefore, the ΔE_ST_ values were obtained based on TD-DFT calculations. Additionally, cyclic monomer structures of PhIN-CPP and ThIN-CPP were extracted as representatives in order to calculate their values, since cyclic structures of the two polymers were too large to be easily calculated using software. The extracted structures are shown in Figure S5 and the calculation results are shown in Figure 10 and Table 3, respectively. It can be seen that PhIN-CPP possessed the smaller ΔE_ST_ values compared to ThIN-CPP, which facilitated the excitation of electrons through intersystem crossing (ISC) for S1 ↔ T1 transfer, and prolonged the lifetime of photo-generated electrons in several organic semiconductors [84]. Additionally, PhIN-CPP possessed a smaller reorganization energy (λ_e_) in comparison to that of PhIN-CPP. Both the smaller ΔE_ST_ and λ_e_ values facilitated the acceleration of electronic transmission, thereby shortening the electron traveling time to the reaction center for HER, which ultimately led to improved photocatalytic activities. Therefore, the interactions between S1 and T1 states were investigated using TD-DFT of the excited states (S1, T1–T3) for PhIN-CPP and ThIN-CPP, and the results are illustrated in Figure 10, and Tables S1 and S2. As shown in Figure 10, the T1 state of PhIN-CPP demonstrated a more pronounced local excitation triplet (^3^LE) feature, which was attributed to its highly planar structure. This structure allowed for almost complete delocalization of both the holes and electrons, resulting in a more uniform distribution within the material. In contrast, the T1 state of ThIN-CPP exhibited a weaker manifestation of locally excited triplet states (^3^LE) characteristics, in which both the holes and the electrons were localized on the pyrene fragment. In the end, the DFT and TD-DFT calculations revealed that the incorporation of triazine units in the as-prepared CMPs played a significant role in hydrogen adsorption and was beneficial to the photocatalytic decomposition of H_2_O. Above all, the results of DFT and TD-DFT calculations were consistent with the experimental results of UV–Vis-URS, photocurrent, impedance, and photocatalytic H_2_ evolution experiments.

### 2.7. Photocatalytic Mechanism

**Scheme 1:**
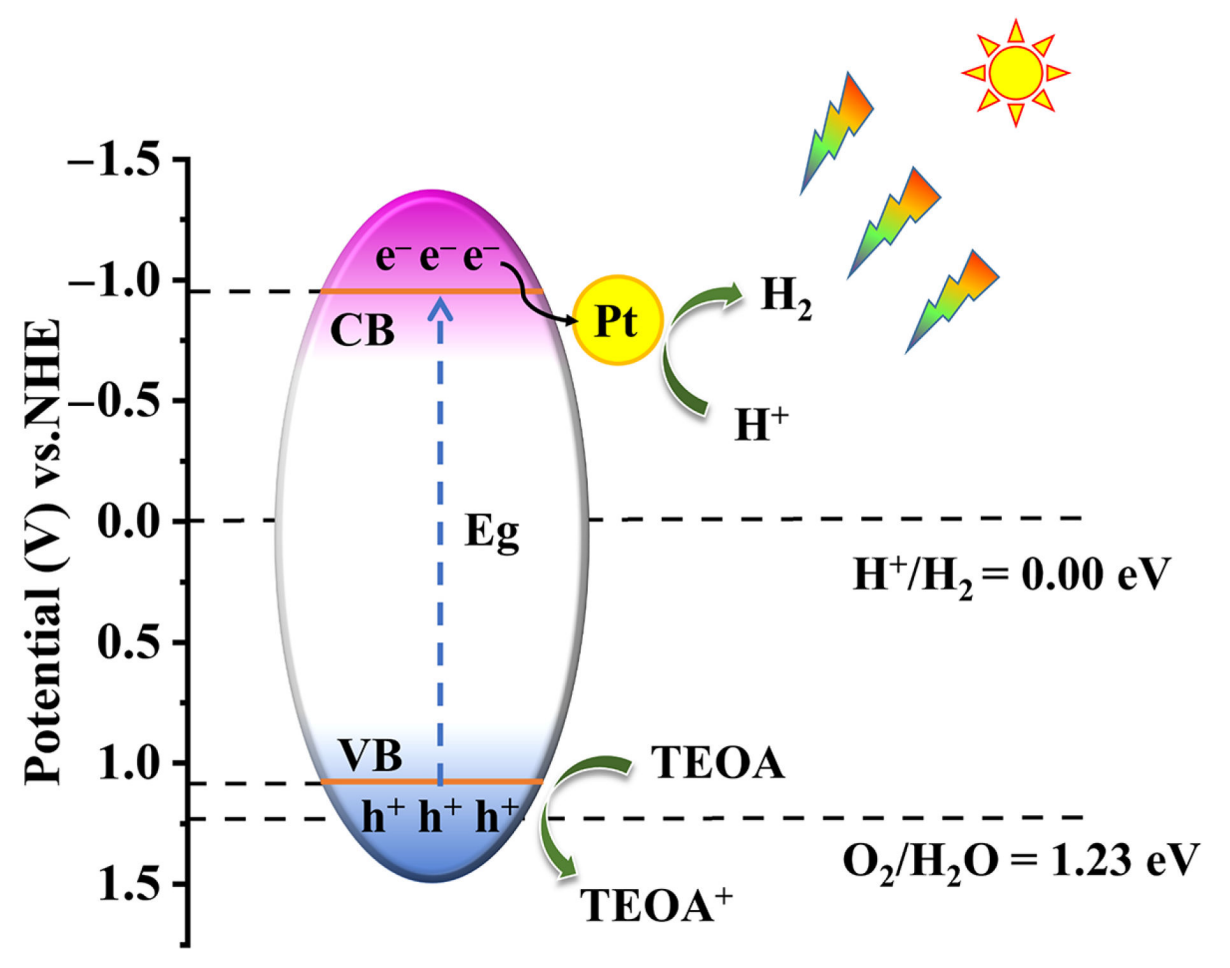
Mechanism of photocatalytic H_2_ production by PhIN-CPP.

Since PhIN-CPP exhibited a higher H_2_ production capacity compared to ThIN-CPP, the hydrogenation mechanism of PhIN-CPP was proposed as representative. As depicted in Scheme 1, PhIN-CPP was capable of absorbing photons under visible light irradiation, which was beneficial to generating electron-hole pairs. Subsequently, the electrons generated by the photo transitioned to the conduction band of the PhIN-CPP. The existence of heterojunctions between the polymer and Pt-catalyst [85] facilitated further transfer of photogenerated electrons from the conduction band of the polymer to the surface of Pt-catalyst. This transfer leads to a reaction with H^+^ in solution, resulting in the production of H_2_. The photogenic hole still persists in the valence band of PhIN-CPP, and it is consumed by the sacrificial agent triethanolamine to produce the corresponding oxidation product. The formation of oxidation products can inhibit the recombination of electrons and holes, thereby improving the efficiency of photocatalytic H_2_ production. This finding suggested that using triethanolamine as a sacrificial agent can enhance the overall performance of PhIN-CPP in photocatalysis. The H_2_ production pathway in equation form is given as follows:

(4) $$\begin{array}{l} H_{2}O\to H^{+}+OH^{-}\\ Photocatalyst+hv\to h^{+}+e^{-}\\ H^{+}+e^{-}\to \cdot H\\ \cdot H+\cdot H\to H_{2}\\ h^{+}+TEOA\to TEOA^{+} \end{array}$$

## 3. Experiments

### 3.1. Materials and Reagents

1-Methyl-2-pyrrolidone (NMP), triethanolamine (TEOA), 5-bromothiophene-2-acetonitrile, chloroform, trifluoromethanesulfonic acid, 2,6-bis(trimethyltin)-dithiopheno[3,2-b;2′,3′-d] thiophene, PdCl_2_(PPh_3_)_2_, chlorobenzene, 2,6-bis(trimethyltin)-dithiopheno[3,2-b;2′,3′-d] thiophene, and methanol were commercially available (Sinopharm Chemical Reagent Co., Ltd., Shanghai, China), and used directly without purification processing.

### 3.2. Synthesis of 2,4,6-tris (5-bromothiophen-2-yl)-1,3,5-triazine

Typically, a Pyrex tube was charged with 5-bromothiophene-2-acetonitrile (4.0 g, 21.3 mmol) and 300 mL of chloroform followed by the addition of 85.2 mmol trifluoromethanesulfonic acid. The mixture was stirred for 2 h at 77 K before the tube was sealed off and heated at ambient temperature for 48 h. The resulting precipitate was collected by filtration, washed with distilled water, and dried using anhydrous magnesium sulfate. Finally, the precipitate was purified through toluene recrystallization to obtain the white solid product with a yield of 87.3%. The synthetic procedure of this monomer is illustrated in Scheme S1 and its ^1^H NMR is shown in Figure S2. The ^1^H NMR (500 MHz, CDCl_3_), δ 7.97 (d, J = 4.0 Hz, 3H), 7.17 (d, J = 4.0 Hz, 3H). ^13^C NMR (126 MHz, CDCl_3_), δ 129.47, 127.46, 127.35, 126.82, 124.74.

### 3.3. Synthesis of PhIN-CPP and ThIN-CPP

**Scheme 2:**
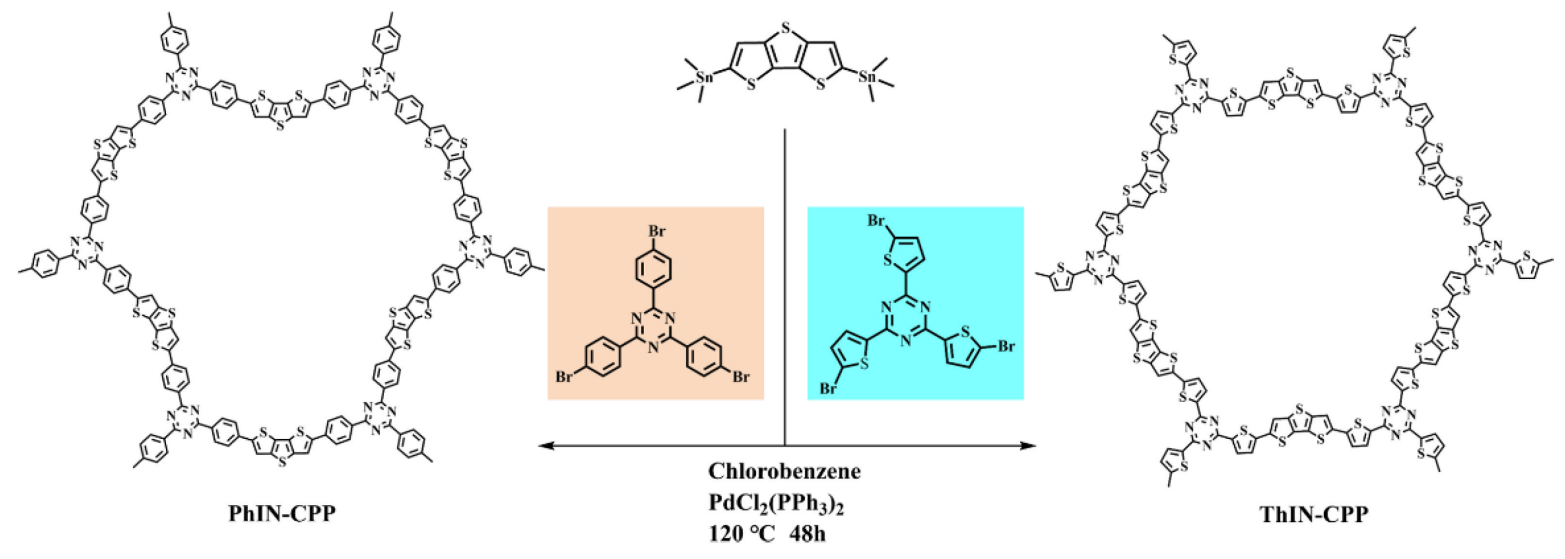
The synthetic procedure of PhIN-CPP and ThIN-CPP.

In this study, PhIN-CPP was synthesized via the Stille coupling reaction under N_2_ atmosphere. The Shrek tube was initially loaded with 2,4,6-tris(4-bromophenyl)-1,3,5-triazine (0.200 g, 0.37 mmol), 2,6-bis(trimethyltin)-dithiopheno[3,2-b;2′,3′-d] thiophene (0.2867 g, 0.55 mmol), PdCl_2_(PPh_3_)_2_ (20 mg), and chlorobenzene (30 mL). The mixture was sonicated for 10 min to achieve a homogeneous dispersion. Subsequently, the Shrek tube was flash frozen at 77 K using a liquid N_2_ bath and degassed through three freeze–pump–thaw cycles. The sealed tube was then heated at 120 °C for 48 h to complete the reaction. The resulting colored precipitate was initially washed with methanol and chloroform before being purified using the Soxhlet extraction method with methanol and chloroform as solvents to remove residual Pd catalysts and soluble oligomers. Each solvent extraction lasted for 24 h. Finally, the precipitate was dried at 70 °C under vacuum for 12 h yielding a product of purity of up to 89.6%. The synthetic process of ThIN-CPP followed a similar procedure to that of PhIN-CPP but substituted 2,4,6-tris (5-bromothiophen-2-yl)-1,3,5-triazine instead of 2,4,6-tris(4-bromophenyl)-1,3,5-triazine. The building-up process for PhIN-CPP and ThIN-CPP is illustrated in Scheme 2.

### 3.4. Characterization

X-ray diffraction (XRD) patterns were obtained using a Philips X’Pert Pro Super diffractometer (Amsterdam, The Netherland) equipped with Cu Kα radiation (λ = 1.54178 Å). The morphologies and compositions of the prepared samples were analyzed using scanning electron microscopy (SEM, JEOL JSM-6700F, Tokyo, Japan) and attached X-ray energy dispersive spectrometry (EDS), respectively. The FT-IR spectra were recorded using a Nicolet-5700 FT-IR spectrophotometer (Thermoelectric Corporation of America, Madison, WI, USA) in the region of 4000 to 400 cm^−1^. Raman spectra were recorded using a LabRam HR Evolution (HORIBA Jobin Yvon, Paris, France). The BET surface area, average pore size, and pore diameter distribution were determined using a Quantachrome Autosorb IQ-C nitrogen adsorption apparatus (McMurdoch Co., Ltd., Norcross, GA, USA) at 77 K. The surface structure and chemical states of the polymers were investigated by X-ray photoelectron spectroscopy (XPS) (Thermo Fisher Scientific, Waltham, MA, USA) with Thermo Scientific ESCALAB 250. The catalyst’s ultraviolet-visible diffuse reflectance spectrum was tested using a Carry 5000 UV–Vis spectrophotometer (Agilent Technologies, Inc., Palo Alto, CA, USA), with a wavelength range of 200–800 nm and BaSO_4_ as the reference. An Edinburgh FI/FSTCSPC 920 spectrophotometer (Edinburgh, Livingston, England) was employed to acquire the photoluminescence spectra (PL).

### 3.5. Photocatalytic H_2_ Evolution Test

The experiment on photocatalytic decomposition of water for H_2_ production was carried out in a Pyrex top-irradiation reactor at room temperature, which was connected to a closed gas system with a glass enclosure. The details of the photocatalysis experiment are described as follows. Firstly, 40 mL H_2_O, 10 mL NMP, and 10 mL TEOA were added into the above reactor, of which TEOA played the role of sacrificial electron donor to prevent the electrons and holes recombining. Then, 30 mg powdered catalyst was uniformly dispersed in the abovementioned solution. The light source used in this study was a 300 W Xeon lamp with an operating current of 15 A (Shenzhen ShengKang Technology Co., Ltd., Shenzhen, China, LX300 F). The gas chromatography accompanied with a thermal conductive detector (TCD) was employed to analyze the evolved gases.

## 4. Conclusions

In summary, two conjugated porous polymers, PhIN-CPP and Thin-CPP, based on triazines, were meticulously designed and successfully synthesized using benzene and thiophene as building blocks. The photonic band gaps of PhIN-CPP and ThIN-CPP were calculated as 2.05 eV and 1.79 eV, respectively. The HER values of PhiN-CPP and ThIN-CPP were 3074.75 and 306.63 μmol·g^−1^·h^−1^, respectively, without any cocatalysts. However, the HER values of PhiN-CPP and ThIN-CPP were up to 5359.92 and 538.49 μmol·g^−1^·h^−1^, respectively, after the addition of 3wt% Pt-catalyst. Notably, PhIN-CPP displayed the highest photocatalytic activity, which can be ascribed to the high planarity of its benzene units, its fluffier structure, larger specific surface area, relatively strong absorption in the visible region, and high conjugated structure (electron richness). This study clearly indicates that polymers formed by condensation of simple monomers can also achieve efficient and stable photocatalytic H_2_ production. The ΔE_ST_ values for PhIN-CPP and ThIN-CPP were calculated as 0.79 eV and 0.80 eV, respectively, based on DFT and TD-DFT calculations, which suggested that incorporation of triazine units in the as-prepared CMPs could accelerate the separation and transfer of electrons upon light absorption and further reduce ΔE_ST_ value. Furthermore, selecting the ideal connection unit to design the molecular structure of triazine-based conjugated porous polymer will be one of the effective ways in the future to construct novel photocatalytic organic materials with high efficiency and a stable hydrogen precipitation rate.

## Figures and Tables

**Table 1:** Photophysical properties and hydrogen precipitation rate of PhIN-CPP and ThIN-CPP.

Polymer	λ_abs_ *^a^* (nm)	Eg *^b^* (eV)	E_VB_ *^c^* (eV)	E_CB_ *^d^* (eV)	HER *^e^* (μmol g^−1^ h^−1^)	HER *^f^* (μmol g^−1^ h^−1^)
PhIN-CPP	630	2.05	1.09	−0.96	3074.75	5359.92
ThIN-CPP	706	1.79	1.15	−0.64	306.63	538.49

*^a^* Light absorption edge. *^b^* The optical band gap calculated according to the absorption edge of the ultraviolet visible diffuse reflection spectrum. *^c^* The valence band value of the polymer relative to the standard hydrogen electrode. *^d^* The conduction value of the polymer relative to the standard hydrogen electrode. *^e^* Photocatalytic hydrogen production rate of 30 mg pure polymer (no added catalyst) under visible light exposure (λ > 420 nm). *^f^* 30 mg polymer (add 3wt% Pt catalyst) photocatalytic hydrogen production rate under visible light exposure (λ > 420 nm).

**Table 2:** The hydrogen evolution rates (HER) of other materials.

Photocatalysts	HER (μmol·g^−1^·h^−1^)	References
g-C_3_N_4_/WS_2_	101	[58]
PBN	223.5	[59]
NDI-BTzF-PS-PEG-COOH	634.6	[60]
PS-Odec	680	[62]
WS_2_-WO_3_·H_2_O/g-C_3_N_4_	1276.9	[61]
1T-WoS_2_@TiO_2_@Ti_3_C_2_	3409.8	[63]
SFBtBT_0.02_	4210	[64]
BT-TT@ZnS	15,800	[65]
ThSo-1	111,790	[66]
Py-TP-BTDO	115,030	[56]
DBC-BTDO-2	301,920	[57]
PhIN-CPP	5359.92	This study
ThIN-CPP	538.49	

**Table 3:** The excited energies (E_S1_ and E_T1_ in eV). The energy difference between S1 and T1 (ΔE_ST_ in eV), excitation wavelength (λ, nm), oscillator strength (f_S1_ in eV) of the S1 state at neutral ground state geometry, and reorganization energy for electron transfer (λ_e_ in eV) for the monomer of PhIN-CPP and ThIN-CPP.

Monomer	E_S1_	E_T1_	ΔE_ST_	λ (nm)	f_S1_	λ_e_
PhIN-CPP	2.62	1.83	0.79	472.43	2.30	0.26
ThIN-CPP	2.38	1.58	0.80	526.76	2.39	0.27

## Data Availability

The data presented in this research are available on request from the corresponding author.

## Supplementary Materials

The following supporting information can be downloaded at: https://www.mdpi.com/article/10.3390/molecules29122807/s1, Scheme S1: The synthetic procedure of 2,4,6-tris (5-bromothiophen-2-yl)-1,3,5-triazine; Figure S1: ^1^H NMR spectrum of 2,4,6-tris (5-bromothiophen-2-yl)-1,3,5-triazine; Figure S2: XRD patterns of PhIN-CPP and ThIN-CPP; Figure S3: PhIN-CPP before and after visible light irradiation (a) UV diffuse reflection spectra, (b) FT-IR, and (c) XRD pattern; Figure S4: (SEM images of PhIN-CPP before and after visible light irradiation; Figure S5: Extracted structures of PhIN-CPP and ThIN-CPP. Table S1: The excited energies from S0 to S1–S10 and S0 to T1–T5 (eV), excitation wavelength (λ, nm), and oscillator strength (f) for extracted structure of PhIN-CPP. The orbitals and their orbital energies (eV) are shown below the table, where the yellow and cyan colors are positive and negative orbital phases whose isovalue was 0.02; Table S2: The excited energies from S_0_ to S_1_–S_10_ and S_0_ to T_1_–T_10_ (eV), excitation wavelength (λ, nm), and oscillator strength (*f*) for ThIN-CPP. The orbitals and their orbital energies (eV) are shown below the table, where the yellow and cyan colors are positive and negative orbital phases whose isovalue was 0.02.
