# Supplementary material for: Triazine and Fused Thiophene-Based Donor-Acceptor Type Semiconducting Conjugated Polymer for Enhanced Visible-Light-Induced H2 Production
Source: Molecules. 2024 Jun 12;29(12):2807. doi: 10.3390/molecules29122807 (PMC11206750; doi:10.3390/molecules29122807)
Supplement: Supplementary file 1 [file molecules-29-02807-s001.zip › molecules-3037137-supplementary.pdf]

Supplementary Material

Triazine and Fused Thiophene-Based Donor-Acceptor Type Semiconducting Conjugated Polymer for Enhanced Visible-Light-Induced H_2 Production

Jian Liu [1,2], Shengling Zhang [3], Xinshu Long [4], Xiaomin Jin [4], Yangying Zhu [4], Shengxia Duan [4,5*], Jinsheng Zhao [3*]

1 College of Agriculture and Bioengineering, Heze University, Heze 274000, China

2 Institute of Biotechnology, Chinese Academy of Tropical Agricultural Sciences, Haikou 570100, China

3 Department of Chemistry and Chemical Engineering, Liaocheng University, Liaocheng 252000, China

4 Department of Chemistry and Engineering, Heze University, Heze 274500, China

5 CAS Key Laboratory of Photovoltaic and Energy Conservation Materials, Institute of Plasma Physics, Chinese Academy of Sciences, Hefei, 230031, China

Corresponding Author

*Tel/Fax: +86-530-5529575. Email: sxduan@ipp.ac.cn; j.s.zhao@163.com.

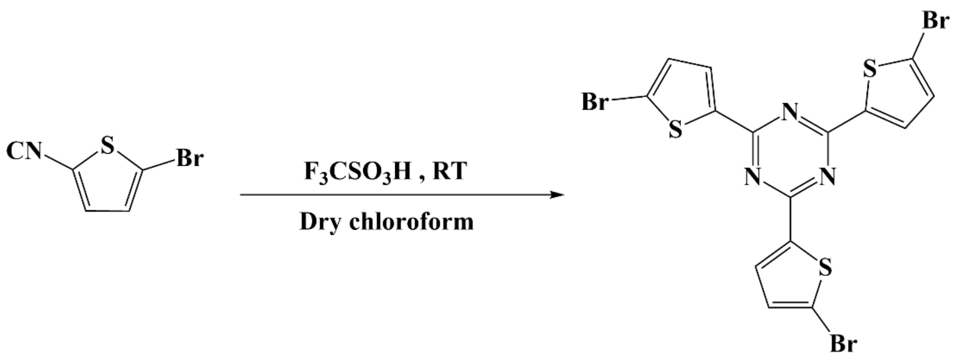

Scheme S1 The synthetic procedure of 2,4,6-tris (5-bromothiophen-2-yl)-1,3,5-triazine.

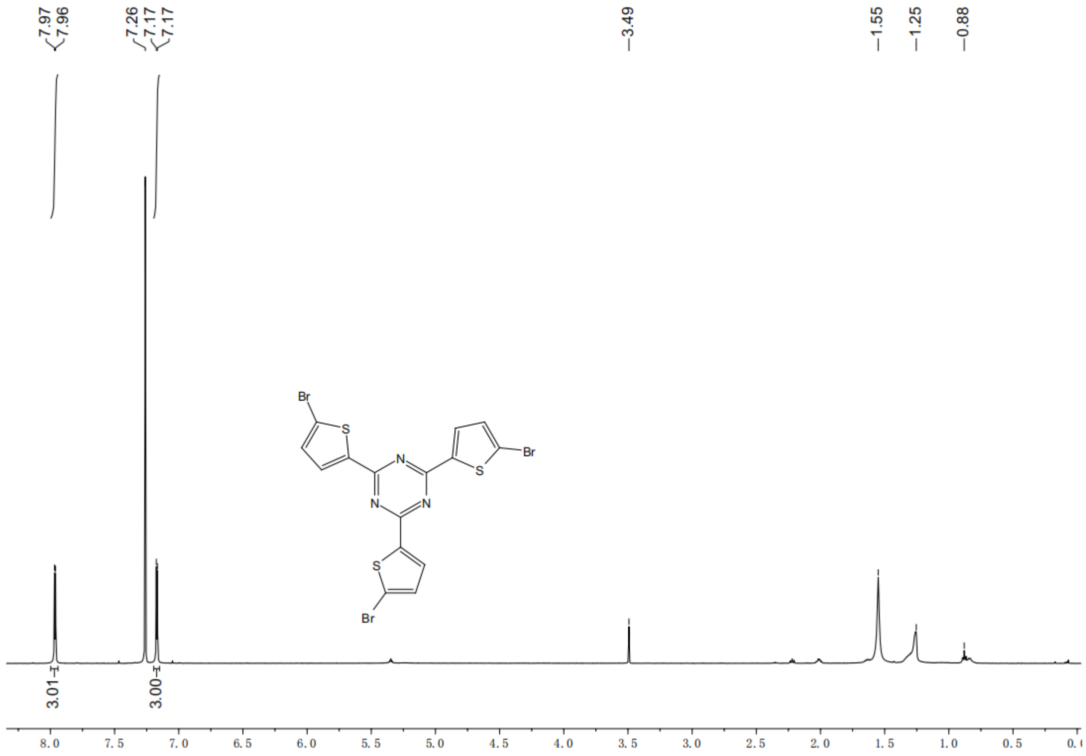

Figure S1 ^{1}H NMR spectrum of 2,4,6-tris (5-bromothiophen-2-yl)-1,3,5-triazine.

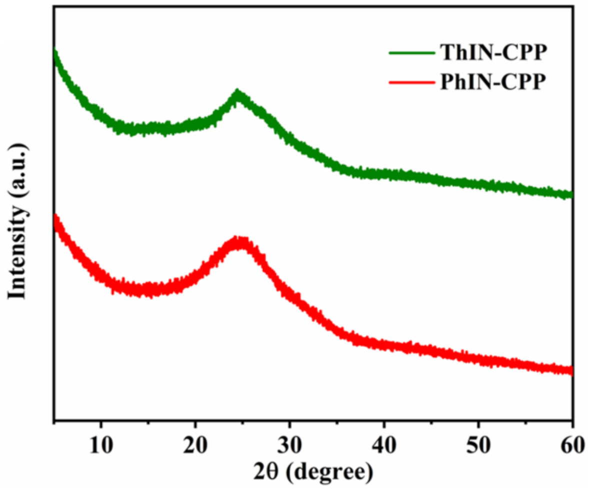

Figure S2 XRD patterns of PhIN-CPP and ThIN-CPP.

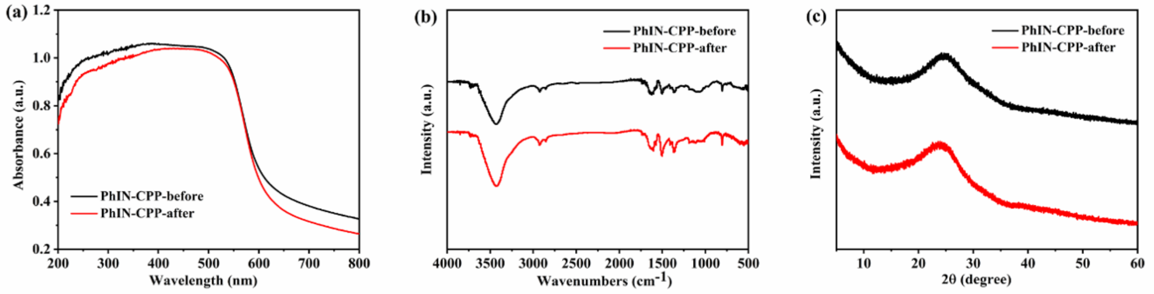

Figure S3 PhIN-CPP before and after visible light irradiation (a) UV diffuse reflection spectra, (b) FT-IR and (c) XRD pattern.

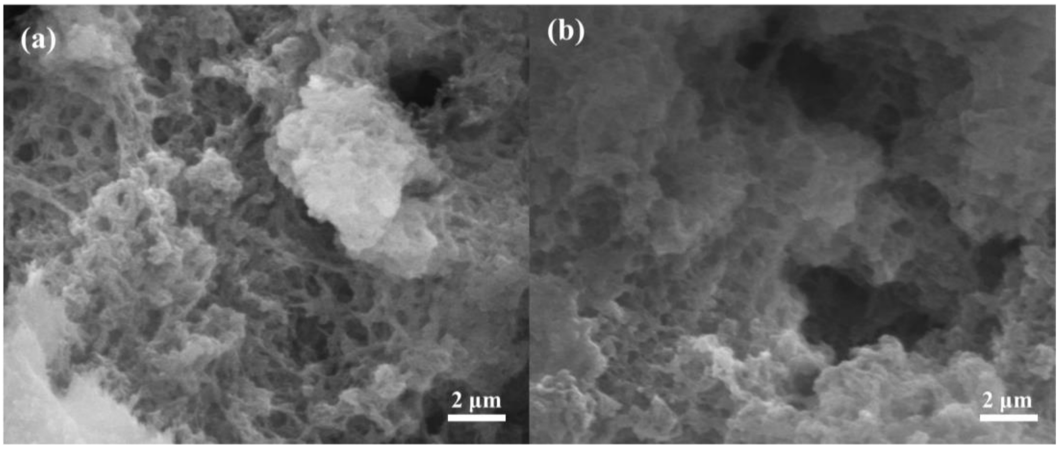

Figure S4 SEM images of PhIN-CPP before and after visible light irradiation.

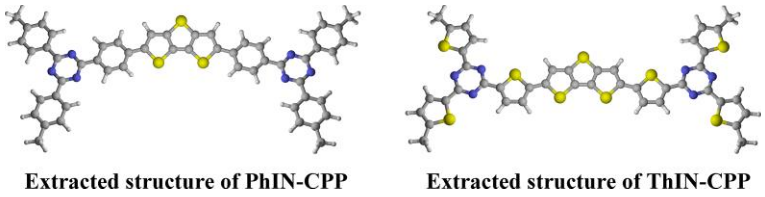

Figure S5 Extracted structures of PhIN-CPP and ThIN-CPP.

Table S1 The excited energies from S0 to S1-S10 and S0 to T1-T5 (eV), excitation wavelength (λ, nm), and oscillator strength (f) for extracted structure of PhIN-CPP. The orbitals and their orbital energies (eV) were shown below the table, where the yellow and cyan colors are positive and negative orbital phases whose isovalue was 0.02.

State	Excitation	E	λ(nm)	*f*
S1	H -> L 98.1%	2.6244	472.43	2.29630
S2	H -> L+1 96.8%	3.0903	401.20	0.03560
S3	H -> L+2 97.8%	3.1404	394.80	0.00000
S4	H -> L+3 97.6%	3.1407	394.77	0.00010
S5	H-1 -> L 98.1%	3.3829	366.50	0.01000
S6	H -> L+4 95.5%	3.6913	335.88	0.12570
S7	H-3 -> L 78.0%<br>H-4 -> L+1 13.6%	3.7428	331.26	0.00510
S8	H-4 -> L 78.4%<br>H-3 -> L+1 13.7%	3.7435	331.20	0.05520
S9	H-1 -> L+1 96.8%	3.8061	325.75	0.04090
S10	H-2 -> L 86.3%	3.8181	324.73	0.06730

T1	H -> L 85.4%<br>H -> L+4 8.0%	1.8331	676.36	0.0000
T2	H -> L+1 61.0%<br>H-2 -> L 18.7%<br>H -> L+5 7.0%	2.4494	506.18	0.0000
T3	H-1 -> L 85.6%<br>H-1 -> L+4 7.9%	2.9422	421.40	0.0000
T4	H-4 -> L+3 19.4%<br>H-3 -> L+2 19.3%<br>H -> L+4 12.2%<br>H-2 -> L+1 11.8%<br>H-5 -> L 6.4%	2.9502	420.26	0.0000
T5	H-3 -> L+3 27.6%<br>H-4 -> L+2 27.6%<br>H-5 -> L+1 8.0%	3.0079	412.20	0.0000

Table S2 The excited energies from S_0 to S_1-S_{10} and S_0 to T_1-T_{10} (eV), excitation wavelength (λ, nm), and oscillator strength (f) for ThIN-CPP. The orbitals and their orbital energies (eV) were shown below the table, where the yellow and cyan colors are positive and negative orbital phases whose isovalue was 0.02.

State	Excitation	E	λ(nm)	f
S1	H -> L 97.7%	2.3837	526.76	2.39240
S2	H -> L+1 94.0%	2.8347	437.38	0.00010

S3	H -> L+2 96.8%	2.8863	429.56	0.00000
S4	H -> L+3 96.2%	2.8869	429.47	0.00030
	H-1 -> L 74.4%			
S5	H-6 -> L 13.3%	3.2538	381.04	0.02730
	H-5 -> L 5.5%			
	H-3 -> L 80.3%			
S6	H-2 -> L+1 9.7%	3.2788	378.14	0.00150
	H-2 -> L 80.0%			
S7	H-3 -> L+1 9.2%	3.2799	378.01	0.03070
	H-4 -> L 85.3%			
S8	H-5 -> L+1 6.0%	3.3515	369.94	0.00080
	H-5 -> L 85.6%			
S9	H-4 -> L+1 5.9%	3.3534	369.73	0.00280
S10	H -> L+4 93.0%	3.4383	360.60	0.09440
T1	H -> L 88.9%	1.5790	808.98	0.0000
	H -> L+1 57.4%			
T2	H-1 -> L 23.0%	2.0565	602.89	0.0000
	H-6 -> L 7.6%			

T3	H -> L+5 5.9%<br>H-2 -> L+3 27.4%<br>H-3 -> L+2 27.0%<br>H-4 -> L+4 7.9%<br>H-5 -> L+1 7.2%<br>H-4 -> L 6.9%<br>H-5 -> L+5 6.4%	2.5502	486.17	0.0000
T4	H-2 -> L+2 29.3%<br>H-3 -> L+3 29.2%<br>H-4 -> L+1 8.2%<br>H-5 -> L+4 8.1%<br>H-4 -> L+5 6.8%	2.5717	482.11	0.0000

	H-5 -> L 6.6%
